# Genome-Wide Analysis of Dynamin Gene Family in cassava (*Manihot esculenta Crantz*) and Transcriptional Regulation of Family Members *ARC5* in Hormonal Treatments

**DOI:** 10.3390/ijms20205094

**Published:** 2019-10-14

**Authors:** Peng Cao, Xiaohan Liu, Jianchun Guo, Yinhua Chen, Shuangbao Li, Congcong Wang, Wu Huang, Yi Min

**Affiliations:** 1 Key Laboratory of Tropical Biological Resources of Ministry of Education, College of Life Sciences and Pharmacy, Hainan University, Haikou 570228, China; 17095103210001@hainanu.edu.cn (P.C.); 17071008210002@hainanu.edu.cn (X.L.); yhchen@hainanu.edu.cn (Y.C.); 18071008210002@hainanu.edu.cn (S.L.); 16071008210003@hainanu.edu.cn (C.W.); 16095103210001@hainanu.edu.cn (W.H.); 2 Institute of Tropical Bioscience and Biotechnology, Chinese Academy of Tropical Agricultural Sciences, Haikou 571101, China; guojianchun@itbb.org.cn

**Keywords:** plastid division, dynamin, *ARC5*, duplication event, expression, hormone treatment, cassava

## Abstract

The Dynamin gene family play a significance role in many physiological processes, especially *ARC5* (Accumulation and replication of chloroplasts 5) in the process of plastid division. We performed a genome-wide analysis of the cassava Dynamin family based on the published cassava genome sequence and identified *ARC5*. 23 cassava Dynamins (*MeDynamins*) were identified and renamed. 23 *MeDynamins* were further divided into five major groups based on their structural and phylogenetic characteristics. The segmental duplication events have a significant impact on the expansion of MeDynamins. *ARC5* expression analysis showed that there were differences between leaves and roots of cassava at different developmental stages. The tissue-specific expression analysis of the MeDynamins showed that most of MeDynamins were expressed in stem apical meristem and embryogenesis, whereas ARC5 was mainly expressed in leaves. The processing of IAA (Indole-3-acetic Acid) and MeJA (Methyl Jasmonate) verified the prediction results of cis-elements, and ACR5 was closely related to plant growth and positively correlated. It also indicated that high concentrations of MeJA treatment caused the cassava defense mechanism to function in advance. In conclusion, these findings provide basic insights for functional validation of the ARC5 genes in exogenous hormonal treatments.

## 1. Introduction

Plastids descended from a free-living cyanobacterium, which formed a chloroplast in a potentially not too long historical period through an endosymbiotic event more than a billion years ago, and then evolved into the different types of plastids that we now see [1,2]. Chloroplasts are chlorophyll-containing plastids, which are ubiquitous in green plants and are the places where green plants photosynthesize. Chloroplasts are plant-specific organelles, and their main mode of proliferation is binary division. The division of chloroplasts is achieved by the synergistic action of various proteins. These proteins have both prokaryotic origin, such as FtsZ (the filamentous temperature sensitive protein Z) family and ARC6 [3,4], and eukaryotic origin, such as PDV1 (plastid division1), PDV2 and ARC5 [5,6]. The previous study showed that the majority of genes regulating cyanobacterial cell division undergo metastasis and translation after endosymbiosis [7], but that other genes of eukaryotic origin have been recruited to function in plastid division [8,9,10]. The most prominent gene is ARC5 in this process, a GTPase-induced actin, also called DRP5B (Dynamin-related protein 5B) [11].

ARC5 is recruited to the cleavage site of the outer membrane of the chloroplast under the action of PDV1 and PDV2 to form a dynamic ring [5], which acts as pivotal part in the contraction of the chloroplast division [12]. It also plays an additional role in peroxisome division [13]. Studies had shown that after ARC5 was knocked out, the number of chloroplasts was remarkably decreased, the volume was significantly increased, and the shape was often dumbbell-shaped [14,15]. 

ARC5 is one of the GTPase eukaryotic Dynamin family. The Dynamin family participate in a variety of biological processes, such as mitochondria and peroxisome division, endocytosis, cell plate formation, cytokinesis, and intracellular vesicle trafficking [16,17,18,19,20,21]. Other studies indicated that members of the Dynamin family also function as molecular switches, similar to the classical signal GTPase [22]. A common feature of all dynamins and dynamin-related proteins is the presence of a GTPase effector domain (GED), a middle domain (MD), and a large N-terminal GTPase domain [23].

Cassava (*Manihot esculenta*) is a perennial crop that originates from South America [24]. It grows mainly with its tuberous roots and has strong drought tolerance and high adaptability to poor soils [24,25,26]. As a high-yielding starch crop, cassava has attracted more and more people’s attention and is suitable for the production of first- and second-generation bioethanol [27,28]. It is also used in chemical, food, pharmaceutical, paper, and textile industries [29,30,31]. The cost of cassava production is lower than that of grain feed. Cassava roots, stem, and leaf can be used as feed for livestock and poultry to deal with the problem of insufficient forage grass and shortage of coarse roughage [32]. The process of plastid division affects the quality and yield of cassava, so the study of plastid division genes can provide new ideas for the improvement of cassava varieties. It would be necessary to systematically investigate the *ARC5* gene and the Dynamin family in cassava while using the availability of the cassava genome due to the significance of the *ARC5* gene in plastid division.

## 2. Results

### 2.1. Identification of the Dynamin Proteins in Cassava 

A total of 23 putative Dynamin genes were identified in the cassava genome through a BLAST search and HMMER analysis and were annotated as the MeDynamins, and 23 MeDynamins were renamed from MeDynamin1 to MeDynamin23 according to their order in which they were screened. Tables S1 and S2 summarizes gene characteristics for the 23 MeDynamins. Among them, MeDynamin6 was identified as the smallest protein with 262 amino acids (aa), while the largest proteins were MeDynamin3 and MeDynamin11 (925aa). The MW of the proteins ranged from 5.22 (MeDynamin3) to 9.21 (MeDynamin14). The predicted subcellular localization indicated that six MeDynamins were positioned in mitochondrial matrix space, five proteins were positioned in microbodies (peroxisomes), two proteins were positioned in cytoplasm, five proteins were positioned in chloroplast matrix, and one protein was positioned in plasma membrane. One protein was positioned in the endoplasmic reticulum (membrane), three proteins were positioned in the nucleus, and MeDynamin23 was shown to be localized in the nucleus. 

### 2.2. Multiple Sequence Alignment, Phylogenetic Analysis, and Classification of MeDynamins

**Figure 1:**
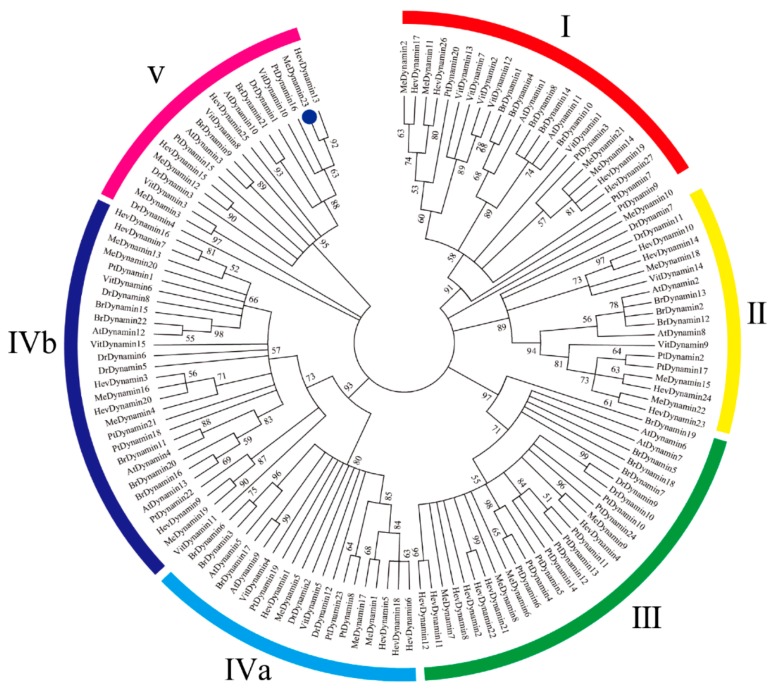
Unrooted phylogenetic tree representing relationships among Dynamin domains of six species. The different-colored arcs and roman numerals indicate different groups (or subgroups) of Dynamin domains. The blue solid circles represent ARC5 from cassava.

An unrooted phylogenetic tree was constructed to analyze the evolutionary relationships of *Dynamin* genes in cassava, *Arabidopsis thaliana*, *Populus trichocarpa*, *Brassica rapa*, *Hevea*, *Vitis vinifera,* and *Dioscorea rotundata* (Figure 1 and Table S3). 136 Dynamins (13 *Arabidopsis thaliana*, 24 *Populus trichocarpa*, 22 *Brassica rapa*, 27 *Hevea*, 15 *Vitis vinifera,* and 12 *Dioscorea rotundata*) were divided into five large groups. Among them, Group I contained 24 Dynamins, four MeDynamins (MeDynamin2,11,14 and 21), together with two AtDynamins from Arabidopsis thaliana, four PtDynamins from *Populus trichocarpa*, five BrDynamins from *Brassica rapa*, four HevDynamins from *Hevea brasiliensis* and five VitDynamins from *Vitis vinifera* were attributed to Group I. Group II consisted of three MeDynamins (MeDynamin15,18, and 22), four HevDynamins, two VitDynamins, two AtDynamins, three BrDynamins, two PtDynamins, and two DrDynamins from *Dioscorea rotundata*. Four MeDynamins (MeDynamin6,7,8 and 9) clustered with two AtDynamins, two DrDynamins, nine PtDynamins, four BrDynamins, and seven HevDynamins in Group III. According to the degree of branching of the phylogenetic tree, Dynamin members in group IV can be further integrated into two subgroups (IVa and IVb), group IVa contained three MeDynamins (MeDynamin1,5 and 17), two AtDynamins, two DrDynamins, two VitDynamins, three BrDynamins, two PtDynamins, and four HevDynamins. Group IVb contained five MeDynamins (MeDynamin4, 13,16,19, and 20), three AtDynamins, three DrDynamins, three VitDynamins, five BrDynamins, four PtDynamins, and five HevDynamins. Group V consisted of three MeDynamins (MeDynamin3,12, and 23), two AtDynamins, two DrDynamins, three HevDynamins, three VitDynamins, two BrDynamins, and two PtDynamins. MeDynamin10 and DrDynamin4 were excluded from the phylogenetic tree because it was too divergent to be aligned with other sequences. Moreover, a total of four sister pairs of MeDynamins were observed in the phylogenetic tree, including MeDynamin1-MeDynamin7, MeDynamin6-MeDynamin8, MeDynamin13-MeDynamin20, and MeDynamin14-MeDynamin21. Figure S1 illustrates a multiple sequence alignment of the MeDynamins, where the black frame circles the parts that differs from the conserved sequence. Further phylogenetic and the protein architecture analysis validated the above grouping statement (Figure S2).

### 2.3. Gene Structure Features of MeDynamins

**Figure 2:**
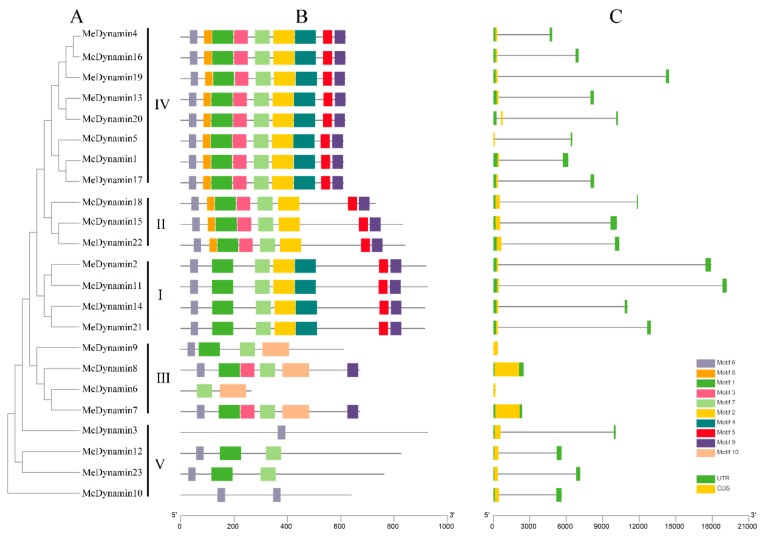
Phylogenetic relationships, gene structure and architecture of conserved protein motifs in *Dynamin* genes from cassava. (**A**) The phylogenetic tree was constructed based on the full-length sequences of cassava dyanmain proteins using MEGA 6 software. (**B**) The motif composition of cassava Dynamin proteins. The motifs, numbers 1–10, are displayed in different colored boxes. The sequence information for each motif is provided in Supplementary Table S4. (**C**) Exon-intron structure of *MeDynamins*. Red boxes indicate untranslated 5′- and 3′-regions; yellow boxes indicate CDS (Coding sequences); black lines indicate introns. The length of protein can be estimated while using the scale at the bottom.

The 10 conserved motifs of MeDynamins were identified by the MEME program. The length ranges from 29 to 100 amino acids. As shown in Figure 2A,B, in addition to motif 6 of the widely distributed MeDynamins domain (lack of MeDynamin3 and MeDynamin6), it was generally found that MeDynamins within the same group shared a similar motif composition (Table S4). For example, motif 10 is unique to group IV, motifs 3 and 8 are specific for groups I and II, motif 4 is specific for groups I and III, and motif 7 is specific for groups II, II, III and IV, motif 1 is specific for groups I, II, and IV (except MeDynamin9 and MeDynamin6), motifs 2, 5, and 9 are specific for groups I, II, and III, but MeDynamin18 has no motif 9. Glycine motif was discovered in MeDynamin23 belonging to group v, which increased plant chlorophyll content and increased enzyme activity, and MeDynamin23 was characterized as the G protein-Dynamin gene in cassava. 

The exon-intron structure of *MeDynamin*s were examined to obtain a deeper understanding of the evolution of the Dynamin family in cassava. All *MeDynamin*s only have one exon (Figure 2C). Among the *MeDynamin*s, only one has two introns (*MeDynamin20*), two have no introns (*MeDynamin7* and *MeDynamin8*), and two have only cds (*MeDynamin6* and *MeDynamin9*). Genes within the same group usually have similar structures, such as *MeDynamin12* and *MeDynamin23,* are observed to be structurally similar.

### 2.4. Promoter Analysis

**Figure 3:**
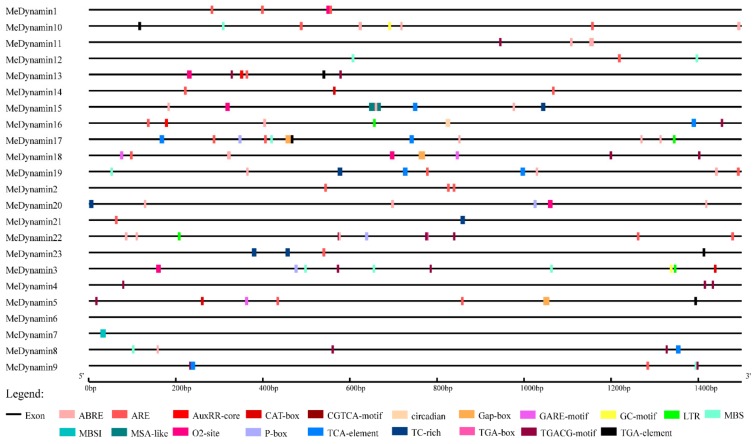
Analysis of *cis*-elements in the 5′-flanking sequences of *MeDynamins*. A total of 20 putative *cis*-elements are represented by different symbols as indicated in various colors. Relative positions of the 20 motifs are marked along the 1.5- kb 5′-flanking sequences of each *MeDynamin.*

We analyzed 20 *cis*-elements, including 17 hormones and stress-related cis-elements, including e.g., abscisic acid (ABA), salicylic acid (SA), low temperature, and drought, among others, to focus on the effects of cis-elements on members of *MeDynamins*. Others cis-elements were CAT-box (cis-acting regulatory element related to meristem expression), MSA-like (it is involved in cell cycle regulation) and circadian (it involved in circadian control) (Figure 3, Tables S5 and S6). All *MeDynamins* had cis-elements in their promoter regions (except *MeDynamin6*). The ABRE element was present in 10 (43%) promoter sequences that were involved in abscisic acid responsiveness. 57% (13/23) of the *MeDynamins* promoter region had ARE element (it is essential for anaerobic induction). The promoter regions of the *MeDynamins* enriched in different numbers and types of cis elements. For instance, *MeDynamin17* has 8 cis-elements that are evenly positioned throughout the sequence, including the *cis*-element ABRE, ARE, LTR (it is involved in low-temperature responsiveness), MBS (MYB binding site involved in drought-inducibility), P-box (gibberellin-responsive element), TCA-element (it is involved in salicylic acid responsiveness), the TGA-element(auxin-responsive element), indicating that abscisic acid, hypoxia, hypothermia, drought, gibberellin, auxin, and salicylic acid may affect the expression of *MeDynamin17*. *MeDynamin23* had ARE, TC-rich repeats (participation in defense and stress response) and TGA-element, *MeDynamin3* and *5*have CAT-box, which indicates that these features may enable plants to respond to a variety of internal and external stimuli or affect plant tissue-specific expression.

### 2.5. Chromosome Distribution and Synteny Analysis of MeDynamins Gene

**Figure 4:**
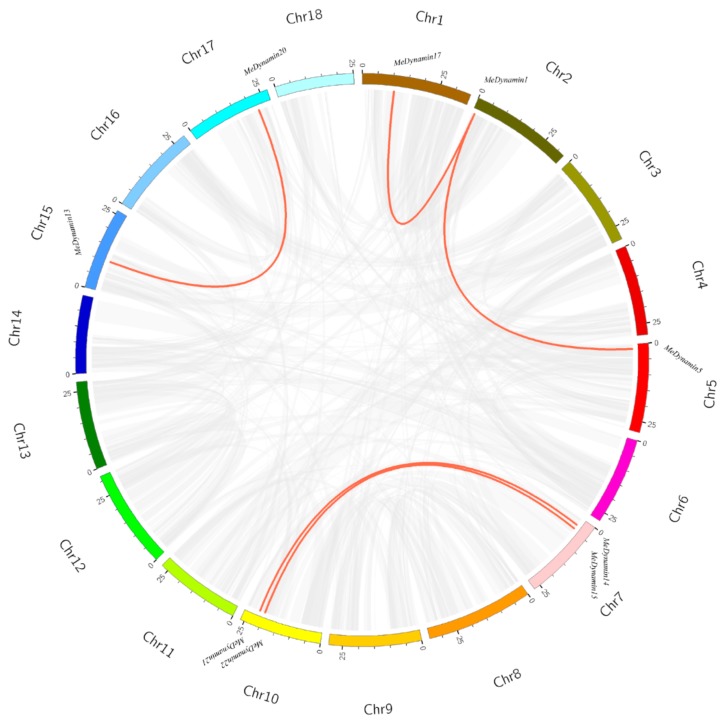
Schematic representations for the chromosomal distribution and interchromosomal relationships of *MeDynamins*. Gray lines indicate all synteny blocks in the cassava genome, and the red lines indicate duplicated Dynamin gene pairs. The chromosome number is indicated at the bottom of each chromosome.

23 *MeDynamin*s were unevenly distributed on 11 chromosomes (Figure S3). The majority of *MeDynamin*s were positioned on n the proximal or distal ends of the chromosomes. There is only one gene on chromosome 6 and chromosome 18, and *MeDynamin23* is located at the end of the chromosome 10. As shown in Figure 4, we identified 10 segmental duplication events with 23 *MeDynamin*s while using the BLASTP and MCScanX methods (Tables S7 and S8). The results indicated that some *MeDynamin*s may be produced by gene duplication, and segmental duplication events are a major driving force for MeDynamins evolution. 

**Figure 5:**
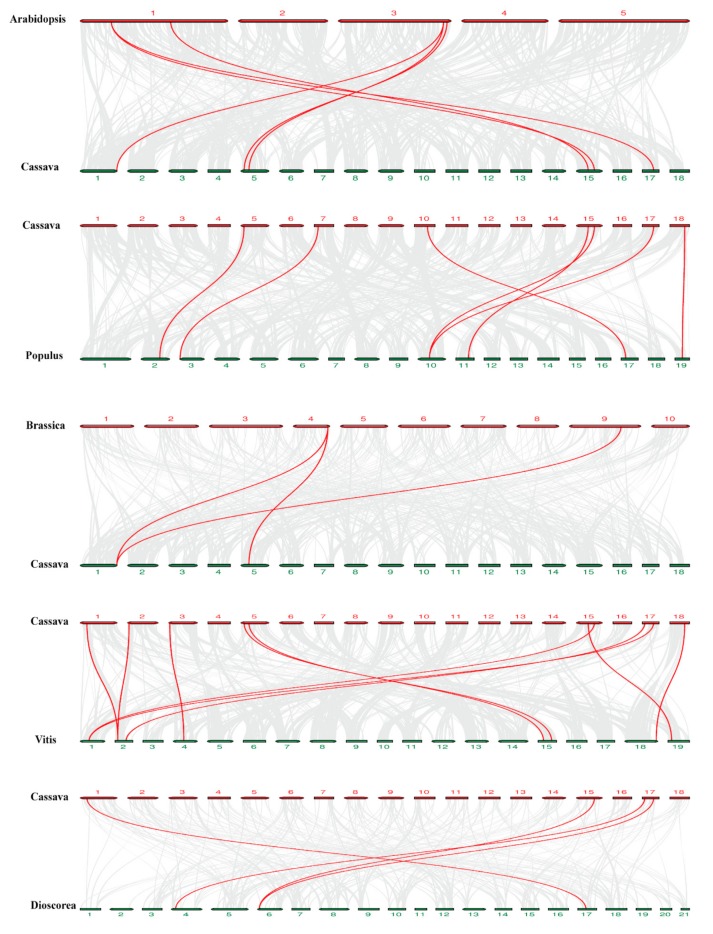
Synteny analysis of *Dynamin* genes between cassava and five representative plant species. Gray lines in the background indicate the collinear blocks within cassava and other plant genomes, while the red lines highlight the syntenic *Dynamin* gene pairs.

We generated five comparative syntenic maps of cassava related to five species, including *Arabidopsis thaliana*, *Populus trichocarpa*, *Brassica rapa,* and *Vitis vinifera* and *Dioscorea rotundata* to further deduce the phylogenetic mechanisms of *MeDynamins* gene family (Figure 5). A total of 10 *MeDynamins* showed syntenic relationship with the *Vitis vinifera* gene, followed by *Populus trichocarpa* (7), *Arabidopsis thaliana* (6), *Dioscorea rotundata* (4), and *Brassica rapa* (3) (Table S9). Some *MeDynamin*s were found to be associated with at least two syntenic gene pairs, and it is supposed that these genes may have played a significant role in the MeDynamin family during evolution. Some collinear gene pairs existed in cassava and other species together (with three *MeDynamin*s), such as *MeDynamin13* / *DrDynamin8*, and *MeDynamin13* / *VitDynamin6*. 

### 2.6. Expression Patterns of MeDynamins in Different Tissues and Developmental Stages

**Figure 6:**
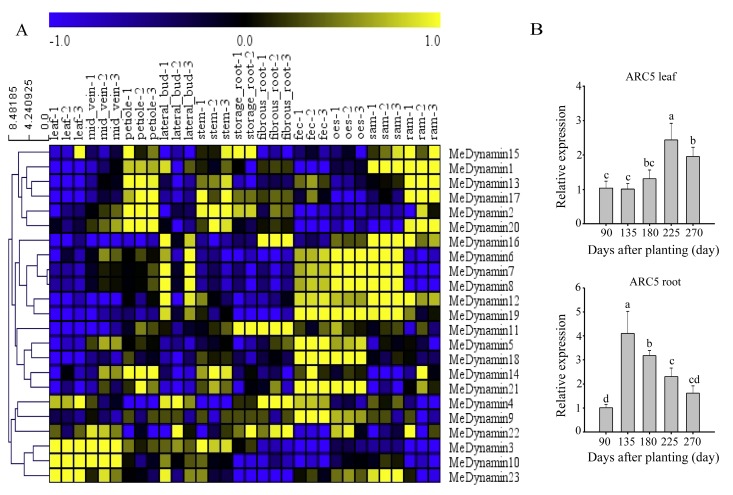
Expression profiles of the cassava Dynamin genes. (**A**) Hierachical clustering of expression profiles of cassava Dynamin genes in different tissues and developmental stages. (**B**) Expression analysis of ARC5 gene in different days by qRT-PCR. Data were normalized to Tubulin gene and vertical bars indicate standard deviation. Small letters (a–d) denote significant variation (*p* < 0.05).

We evaluated their expression data in 11 tissues and organs gained from published GSE82279 to analyze the expression profiles of *MeDynamins* (Table S10) [33]. The 11 tissues were divided into three categories: aerial (leaf, midrib, petiole, stem, lateral bud, and stem apical meristem (SAM)), underground (storage root, fibrous root, and root apical meristem (RAM)), and embryogenesis (organized embryogenic structures and friable embryogenic callus). The results showed that all *MeDynamins* were expressed to at least in one tissue (see also Figure 6). Some *MeDynamins* showed similar expression patterns in various tissues. *MeDynamin1*, *MeDynamin2*, *MeDynamin6*, *MeDynamin7*, *MeDynamin8*, *MeDynamin12*, *MeDynamin13*, *MeDynamin16*, *MeDynamin17*, *MeDynamin19,* and *MeDynamin20* exhibited relatively high expression levels in apical meristem, for instance, SAM and RAM. *MeDynamin5*, *MeDynamin9*, *MeDynamin14*, *MeDynamin18,* and *MeDynamin21* were highly expressed in embryogenesis, *MeDynamin4* and *MeDynamin22* exhibited high expression levels in fibrous root, *MeDynamin15* and *MeDynamin19* showed higher expression levels in storage roots, and other *MeDynamins* were relatively low. *MeDynamin3*, *MeDynamin10,* and *MeDynamin23* were highly expressed in leaves, of which *MeDynamin23* exhibited a quite moderate expression in midrib and SAM. 

Meanwhile, the expression of the *ARC5* gene in cassava leaves and roots was examined by qRT-PCR in different developmental stages after planting (Figure 6). At 90 days after planting, the expression levels of leaves and roots were basically the same, but a great contrast was formed at 135 days after planting. The expression of roots increased sharply to the peak, which was nearly four times higher than the expression of leaves at the same stage, it then declined slowly over time. Leaves expression slowly increased during the amplification stage (135, 180, and 225 days), reached the peak at 225 days, and then decreased at the mature stage, which was similar to that in the root at the same stage.

### 2.7. Differential Expression of ARC5 Gene under IAA and MeJA Treatments

**Figure 7:**
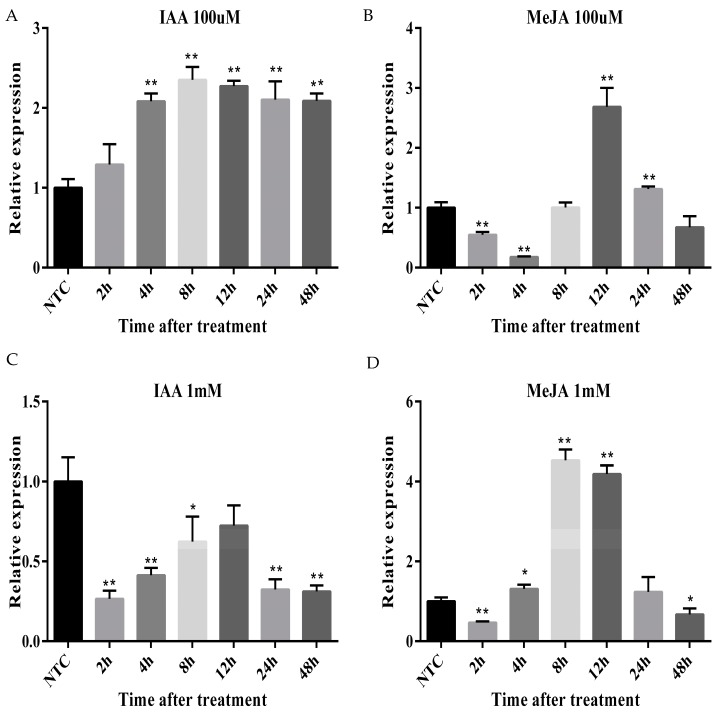
Expression analysis of ARC5 in response to hormonal treatment (**A**) The transcript level of ARC5 under 100 μM IAA; (**B**) The transcript level of ARC5 under 100μM MeJA; (**C**) The transcript level of ARC5 under 1 mM IAA; and, (**D**) The transcript level of ARC5 under 1 mM MeJA. Values are the mean ± SD from three separate experiments. Asterisk (* significant and ** highly significant) denote significant variation (*p* < 0.05).

Cassava seedlings were subjected to IAA and MeJA treatments to investigate the transcriptional responses of *ARC5* gene to hormone (Figure 7). Under 100 μM IAA treatment, *ARC5* transcripts significantly increased with the prolonging of time. The expression level of the treatment group was significantly different from that of the control group during the period from 4 h to 48 h. After the expression level reached the highest peak at 8 h, it was downregulated slowly. Under 100 μM MeJA treatment, *ARC5* transcripts fluctuation changed with the extension of time. At first, the expression of *ARC5* seriously decreased at lowest value at 4 h, and then rose significantly to its peak at 12 h. After that, the expression level sharply dropped and then fell to lower than that of the control group at 48 h. Under 1 mM IAA treatment, the *ARC5* transcripts significantly decreased with the prolonging of time. The expression of *ARC5* plummeted at lowest value at 4h, and then the trend of change was similar to that under 100 μM IAA treatment, however the expression was at the highest peak at 12 h and it was still lower than that of the control group. Under 1 mM MeJA treatment, the *ARC5* transcripts fluctuation changed with the extension of time. Firstly, the expression of *ARC5* had a low valley at 4 h, and then the trend of change was similar to that under 100 μM MeJA treatment, however it reached the highest peak at 8 h rather than at 12 h. The expression of *ARC5* seriously decreased at 24 h and it was roughly the same as that at 4 h. Finally, it was lower than that of the control group at 48 h. These results indicated that the *ARC5* gene showed transcriptional changes under hormonal treatments.

## 3. Discussion

The Dynamin family has only been preliminarily analyzed in *Arabidopsis thaliana* of an annual herbaceous dicot, and species genome-wide analysis has not yet been performed [34]. In this study, the Dynamin family’s genetic structure, gene duplication and protein motif were analyzed in cassava for the first time. Moreover, the expression profiles and subcellular localization assay of *ARC5* were further studied, *Dynamin* genes were screened in cassava genome, and several species were also screened at the same time, such as *Brassica rapa* [35], *Vitis vinifera* [36], *Populus trichocarpa* [37], *Hevea brasiliensis* [38], and *Dioscorea rotundata* [39]. The current study identified 23 MeDynamins, which were renamed as MeDynamin1 to MeDynamin23 based on the selected order, higher than *Arabidopsis thaliana* (13), *Brassica_rapa* (22), *Vitis_vinifera* (15), and *Dioscorea rotundata* (12), but lower than *Populus trichocarpa* (24) and *Hevea brasiliensis* (27). Dynamin proteins are divided into six groups in *Arabidopsis thaliana* [34], but, according to the protein sequence, gene structure, motif, and phylogenetic relationship of cassava, 23 cassava Dynamin proteins were divided into five groups (Figure 1, Figure 2 and Figure S1). In the *Arabidopsis thaliana* phylogenetic tree, AtDynamin3 and AtDynamin10 belong to two subgroups, and, in our study, the genes were all expressed in the V group (AtDynamin10 corresponds to the *ARC5* in *Arabidopsis thaliana*), and the degree of branching of AtDynamin10 and MeDynamin23 in the phylogenetic tree also verified the similarity of genes.

Multiple sequence alignments revealed that the four MeDynamins (MeDynamin3, MeDynamin10, MeDynamin12, and MeDynamin23) in group V have sequence variation in the domain, and, unlike other proteins, there are only a few conserved sequences. The acquisition and loss of domains are different forces of gene family expansion. The loss of domains appears to be common in many monocots, such as rice and maize [40,41,42]. Therefore, these four Dynamin proteins deserve to be further investigated for their function and binding specificity, and the ARC5 (MeDynamin23) studied in this paper belongs to the group V. All proteins (except MeDynamin12 and MeDynamin23, with only one Dynamin domain) had three domains (Dynamin, MD and GED). However, four proteins (MeDynamin2, MeDynamin11, MeDynamin14, and MeDynamin21) had one more Pleckstring homology (PH) domain. In the phylogenetic tree MeDynamin12 and MeDynamin23 belong to the V group, MeDynamin2, MeDynamin11, MeDynamin14, and MeDynamin21 belong to the I group, further verifying the reliability of the phylogenetic tree. 

The close relationship between MeDynamins and HeDynamins was obtained in the phylogenetic tree, reflecting the fact that both cassava and *Hevea brasiliensis* are members of the *Euphorbiaceae*, and this observation also shows that different phylogenetic tree groups exhibit different gene structure features. This finding was also observed in other species, with the development tree-motif of six species as an example (Figure S2), there are 10 motifs in total, which more comprehensively verifies the similarity. The MeDynamin family also had a total of 10 motifs. Similar motifs arrangement in MeDynamins within subgroups indicated that the protein structure is conserved in a particular subfamily. Most of these conserved motifs functions remain to be elucidated. In conclusion, gene structure features of the members in the same group, as well as phylogenetic analysis results, strongly support the reliability of group classification. Similar to motif analysis, all of the *MeDynamin* genes have only one exon, and genes within the same group frequently have similar structures. For instance, *MeDynamin12* and *MeDynamin23* were observed to be structurally similar. Interestingly, most of the MeDynamins were basic proteins with a pI value greater than 7, except for MeDynamin3, MeDynamin7, and MeDynamin8, which have a pI value of less than 6. This might be due to the higher proportion of acidic amino acids, such as glutamate and isoleucine, which are present more than in other MeDynamins.

Gene duplication and divergent events are often considered as essential sources of evolutionary dynamics [43,44]. It also leads to the expansion of gene families, including tandem, fragment, and transfer replication [45]. No tandem duplication occurred in the MeDynamins family, and five pairs of genes were all evolved from fragment duplication (Figure 4), which suggests that fragment duplication might be the primary pattern of duplication for this family expansion. By calculating the Ka/Ks ratio of the Dynamin gene pairs, it was indicated that the cassava Dynamin gene family may undergo intense purification selection pressure during evolution [46]. In addition, some collinear gene pairs existed in cassava and other species together (with three *MeDynamin*s), such as *MeDynamin13* / *DrDynamin8*, *MeDynamin13* / *VitDynamin6*. The ARC5 gene in cassava did not appear in tandem duplication and fragment duplication and it had no collinear relationship with other species, which indiated that ARC5 is highly conserved and has the characteristics of stable inheritance.

Cassava RNA-seq data from NCBI databases were further explored to analyze the expression patterns of MeDynamins in this study. Figure 6 showed that *MeDynamins* exhibited differential expressions in various tissues (for example, *MeDynamin3*, *MeDynamin10,* and *MeDynamin23* were highly expressed in leaves, among which *MeDynamin23* was fairly mild in midveins). This phenomenon has also been supported by the embryonic development of *Arabidopsis thaliana* [34]. In phylogeny, MeDynamin12 was the most closely related to MeDynamin23. However, MeDynamin12 was involved in cytokinesis rather than plastid division [47]. Similarly, two separate families of *Arabidopsis thaliana* dynamin-associated proteins DRP1 and DRP2 are involved in clathrin-mediated endocytosis and cell plate maturation during cytokinesis. DRP2A and DRP2B coordinately function in multiple pathways of post-Golgi trafficking in phosphatidylinositol 3- or 4-kinase and cytoskeleton polymerization-dependent manners [48]. The members of the DRP3-DRP6 family play a role in mitochondria, chloroplast, and peroxisome biogenesis/maintenance and/or no known function [49]. The tissue-specific expression analysis of the *MeDynamin* genes provides evidence of the potential functions of these gene in specific tissues.

In the growth prophase of cassava, the roots draw the nutrients to preferential grow, root cells divide rapidly, and the leaves begin to swell and grow in the anaphase. The color of leaves changed from light green to dark green and the chloroplasts accumulated. The expression of *ARC5* gene in cassava leaves and roots was examined by qRT-PCR in different developmental stages after planting. The expression of ARC5 in leaves significantly increased with the prolonging of time, it was downregulated slowly at 270d. The expression of ARC5 in root seriously increased at 135d, and the expression level then decreased in gradient (Figure 6B). The expression level of MeDynamin23 was low in cassava storage and fibrous root, it was high in cassava leaf and SAM (Figure 6A). The time of the different tissues expression was examined in the late stage of cassava development, and the developmental stage expression of MeDynamin23 in cassava were mutually verified, which illustrates the consistency of expression. It is suggested that higher levels of ARC5 and its homologues may be required for the division of larger chloroplasts during leaf expansion [50]. Studies have shown that the ARC5 homologous protein CmDnm2 in red algae has the function of contracting chloroplasts [51]. Moreover, the function of the *PpDRP5B* is similar to that of DRP5B in *Arabidopsis thaliana*, associated with plastid division in *Physcomitrella patens* [14]. According to the structure of ARC5, the three-dimensional (3D) protein model (Figure S4) and the characteristics of action mechanism, ARC5 provides power for chloroplast contraction and may constitute a motor for PD ring fibril sliding [51]. Therefore, the ARC5 gene exhibiting higher expression in leaves during the growth anaphase might be involved in chloroplast division and leaf growth.

Promoter analysis showed that *MeDynamin23* has many hormone-responsive elements (ARE, TC-rich, and TGA-element), which suggests that the proteins encoded by *MeDynamin23* may function in cassava growth, development and response to environmental conditions. However, promoter analysis is purely based on sequence similarity prediction. To explore whether these cis-elements really function, IAA and MeJA were used to treat cassava in this study. IAA is an auxin that promotes the formation of the top bud tip of plant branches or buds, seedlings, etc. Low concentrations of IAA promote growth, whereas high concentrations of IAA inhibit growth and even cause plant death [52]. Exogenous application of MeJA stimulates the expression of defense plant genes and induces chemical defense of plants. High concentrations of MeJA effectively induce defense responses [53]. In this study, we found that 1 mM IAA significantly inhibited the expression of *ARC5* and 1 mM IAA significantly promoted the expression of *ARC5*. The expression trend of *ARC5* was consistent with the physiological effect of IAA, meanwhile plant hormones and branch-related genes are involved in the regulation of plant branching [54]. *ACR5* was closely related to plant growth and positively correlated. The importance of *ARC5* had been further demonstrated in the process of plastid division. 1 mM MeJA advanced the low and peak of *ARC5* expression. It indicated that high concentrations of MeJA treatment caused the cassava defense mechanism to function in advance. Under MeJA treatment, the expression level of *ARC5* had a tendency to decrease first, increase, and then decrease. 1 mM MeJA significantly increased the expression of *ARC5* and expressed longer than 100 μM MeJA. The previous study showed that high concentrations of MeJA significantly increased CAT, APX, POD, and PPO enzyme activities in sweet potato [53]. Therefore, the processing of IAA and MeJA verified the prediction results of cis-elements.

## 4. Materials and Methods

### 4.1. Identification of Dynamin Genes 

Cassava genome sequence was downloaded from Phytozome database (http://www.phytozome.net/cassava). Local BLAST searches were performed based on the Hidden Markov Model (HMM) profile of Dynamin domains from the Pfam database (PF00350; http://pfam.sanger.ac.uk/) [55,56]. After screening the Dynamin protein sequences, submitting it to CDD (https://www.ncbi.nlm.nih.gov/cdd/), Pfam, and SMART (http://smart.embl-heidelberg.de/) to confirm the conserved Dynamin domain [57]. Length of sequences, molecular weights (MW), isoelectric points (pI), and subcellular location predication for each gene was obtained by using ExPasy (http://web.expasy.org/protparam/) and Psort website (http://psort1.hgc.jp/form.html).

### 4.2. Sequence Analysis and Structural Characterization

The exon-intron structure of *MeDynamins* was determined by comparing the predicted CDSs with their corresponding DNA sequences while using the online Gene Structure Display Server (GSDS: http://gsds.cbi.pku.edu.cn) [58]. The identified conserved Dynamin motifs of cassava were performed by the MEME online program for protein sequence analysis (http://meme.nbcr.net/meme/intro.html) [59], with the following parameters: number of repetitions, any; maximum number of motifs, 10; and, optimum motif length = 6–50 residues. The 3D structural model of ARC5 protein was evaluated using the I-TASSER server (https://zhanglab.ccmb.med.umich.edu/I-TASSER/).

### 4.3. Chromosomal localization and Gene Duplication

The chromosomal location and relative distances of *MeDynamins* were analyzed using MapChart software (https://www.wur.nl/en/show/Mapchart.htm) [60]. Moreover, gene duplication events were analyzed while using the Multiple Collinearity Scan toolkit (MCScanX) with default parameters (http://chibba.pgml.uga.edu/mcscan2/) [61]. The synthetic map of each Dynamin gene duplication segment was generated while using CIRCOS (http://circos.ca/) [62]. The putative duplicated genes were linked by the connection lines. We constructed a homomorphic analysis while using our own python program to demonstrate the homologous relationship of the orthologous *Dynamin* genes acquired from cassava and other species [63]. Non-synonymous (ka) and synonymous (ks) substitution of each duplicated *Dynamin* genes were calculated using KaKs_Calculator 2.0 (KaKs_Calculator2.0 download | SourceForge.net) [64].

### 4.4. Phylogenetic Analysis of Cassava Dynamin Genes Family

The full-length protein sequences of Dynamin from *Arabidopsis thaliana*, *Populus trichocarpa*, *Brassica rapa*, *Dioscorea rotundata*, *Vitis vinifera,* and *Hevea* brasiliensis were based on the descriptions in the relevant literature and they were downloaded from Phytozome database (https://phytozome.jgi.doe.gov/), screened for phylogenetic analysis with Dynamin of cassava. Under the default parameters, 136 complete Dynamin domain cassava genes were multi-aligned and manually aligned while using clustalW [65]. The phylogenetic tree was constructed in MEGA7 by using the Maximum Likelihood method based on the Poisson Model [66,67]. Each node was tested by bootstrap analysis with 1000 replicates. 

### 4.5. Analysis of Cis-Elements in Promoter Region 

The sequence of 1500 bp upstream of each *MeDynamin* gene coding region was screened by our own python program. The cis-elements of promoter regions were screened by PlantCARE (http://bioinformatics.psb.ugent.be/webtools/plantcare/html/).

### 4.6. RNA-Sequencing (RNA-seq) Data Analysis of Dynamin Genes

The RNA-seq data were downloaded from NCBI to study the expression pattern of Dynamin gene in cassava. The RNA-seq data (Table S10) included various developmental stages and tissues. The absolute FPKM (Fragments per kilobase of exon per million fragments mapped) values were divided by the average of all values and the ratios were transformed by log2 to obtain data that are suitable for cluster displays. A heatmap was generated while using the Mev software (https://sourceforge.net/projects/mev-tm4/) [68].

### 4.7. Plant Materials

Cassava cultivar SC8 tissue culture seedling (*Manihot esculenta Crantz* No. SC8) obtained from the Tropical Crops Genetic Resource Institute (TCGRI, Danzhou, China), cultured in the MS rooting medium at 26 °C for a month, would come to be excellent plantlets, and they could be transplanted after seedling adaptation. About two months after planting in pots, two experiments were conducted: (1) To examine the response of cassava to different hormone, including IAA and MeJA treatment, their leaves were collected for qRT-PCR. For IAA treatment, cassava seedlings were watered with 1 mM and 100 μM IAA for 48 h. The treatment of MeJA is consistent with that of IAA [69]. (2) For differential expression analysis of these genes in source and sink organs during tuber root development, the plant materials were collected, as follows: the leaves, tuber phloem and tuber xylem were collected at 90, 135, 180, 225 and 270 days after planting [70]. Three biological samples (different plants) were collected for three technical analyses. The fresh material was used for RNA extraction after freezing in liquid nitrogen and it was preserved in refrigerator at −80 °C.

### 4.8. RNA Extraction and qRT-PCR Analysis

Total RNA from each biological sample was extracted according to the instructions of the manufacturer of TRIquick Reagent (Solarbio, Beijing, China). Using 2 µg of DNase-treated RNA to synthesize the first-strand cDNA, according to the PrimeScript TM RT kit (TaKaRa Biotechnology, Dalian, China). Using SYBR ^®^Premix Ex Taq TM II (TaKaRa Biotechnology, Dalian, China) for qRT-PCR on ABI 7900 Fast Instrument (Applied Biosystems, Foster City, CA, USA). The reactions were performed in a 96-well plate in a volume of 10 μL containing 5 μL 2× SYBR^®^ Premix Ex Taq II (Tli RNaseH Plus), 0.2 μL ROX Reference Dye (50×), 0.4 μL forward and reverse primers (10 μM), 3 μL H2O, 1.2 μL template cDNA (SYBR green reagents were supplied by Takara, Dalian, China). The Applied Biosystems HT7900 apparatus was programmed with the following amplification protocol: 1 min. at 95 °C for one cycle, followed by 40 cycles of PCR (10 s at 95 °C, as well as 15 s at 56 °C and 15 s at 72 °C). The amplification program was followed by a melting curve analysis consisting of 95 °C for 10 s, 65 °C for 60 s, and 95 °C for 5 s. The temperature ramp rate was set to 100% for all steps, except the final ramp between 60 and 95 °C, which was set to 5%. Three technical replicates for each biological sample were analyzed. The relative expression was calculated according to the 2^−ΔΔCt^ method [71]; as an internal control, cassava *Tubulin* gene mRNA was amplified (*Tubulin*-F: 5′ GTTATCCCCTTCCCTCGTCT 3′ and *Tubulin*-R: 5′ TCCTTGGTGCTCATCTTTCC 3′) in an identical manner [70].

### 4.9. Statistical Analysis

Genbank accession number of *ARC5* gene: NC_035170 and *Tubulin* gene: XM_021764783.1. The results in this article were all repeatable. We obtained the Mean values and standard deviations (SDs) from three biological and technical replicates. The t test was used to measure the significant differences among the given treatments. (*p* < 0.05, *n* = 3). The data were presented in graphs and designed while using GraphPad Prism 7.0 (GraphPad Software, Inc., LA Jolla, CA, USA) [72].

## 5. Conclusions

In our study, we have completed basic bioinformatics, such as phylogenetic analysis, exon-intron structure analysis, protein structure prediction, motif analysis, chromosomal localization and gene duplication, multiple sequence alignment, and heat map, to provide comprehensive and basic data for the Dynamin family of cassava. 23 proteins containing Dynamin motif were identified and divided into five groups according to phylogenetic analysis. Most *MeDynamins* were located at the start or end of chromosome. Furthermore, comparisons of the expression levels of *MeDynamin* genes showed that MeDynamins are mainly expressed in stem apical meristem and embryogenesis, whereas *ARC5* was expressed in leaves, which suggested the diverse roles and inimitable transcription levels of Dynamin gene in the cassava plant growth, development, and response to different stresses. The processing of IAA and MeJA verified the prediction results of cis-elements, and *ACR5* was closely related to plant growth and positively correlated. It also indicated that high concentrations of MeJA treatment caused the cassava defense mechanism to function in advance. These outcomes clarify the background for further experiments and provide the basic knowledge to explore the role and possible cross-talk between MeDynamins in plants.

## Supplementary Materials

The following are available online at https://www.mdpi.com/1422-0067/20/20/5094/s1.
