# Supplementary material for: Genome-Wide Analysis of Dynamin Gene Family in cassava (Manihot esculenta Crantz) and Transcriptional Regulation of Family Members ARC5 in Hormonal Treatments
Source: Int J Mol Sci. 2019 Oct 14;20(20):5094. doi: 10.3390/ijms20205094 (PMC6829251; doi:10.3390/ijms20205094)
Supplement: Supplementary file 1 [file ijms-20-05094-s001.zip › ESM_2.doc]

**Figure S1. Protein alignment of the Dynamin genes families.** (a). conserved sequence from 120 to 180; (b). conserved sequence from 220 to 280; (c). conserved sequence from 360 to 440; (d). conserved sequence from 460 to 480.

**Figure S2. Phylogenetic relationships and conserved protein motifs in Dynamin genes from six plant species.** The six species are *Arabidopsis thaliana*, *Brassica rapa*, *Vitis vinifera*, *Dioscorea rotundata*, *Populus trichocarpa* and *Hevea brasiliensis.* The motifs (numbers 1–10) are displayed in different colored boxes. The sequence information for each motif is provided in Supplementary Table 4. The length of protein can be estimated using the scale at the bottom.

**Figure S3. Chromosome distribution of MeDynamins.** MeDynamin23(ARC5) enlarges the mark with navy blue

**Figure S4.** **3‑D conservation structure of ARC5.** (a), (b), (c) and (d) are structural diagrams at different angles.
